# Multifaceted microglia during brain development: Models and tools

**DOI:** 10.3389/fnins.2023.1125729

**Published:** 2023-03-23

**Authors:** Cécile Bridlance, Morgane Sonia Thion

**Affiliations:** ^1^Institut de Biologie de l’École Normale Supérieure (IBENS), École Normale Supérieure, CNRS, INSERM, Université PSL, Paris, France; ^2^Center for Interdisciplinary Research in Biology, Collège de France, CNRS, INSERM, Université PSL, Paris, France; ^3^Collège Doctoral, Sorbonne Université, Paris, France

**Keywords:** microglia, brain, development, models, tools

## Abstract

Microglia, the brain resident macrophages, are multifaceted glial cells that belong to the central nervous and immune systems. As part of the immune system, they mediate innate immune responses, regulate brain homeostasis and protect the brain in response to inflammation or injury. At the same time, they can perform a wide array of cellular functions that relate to the normal functioning of the brain. Importantly, microglia are key actors of brain development. Indeed, these early brain invaders originate outside of the central nervous system from yolk sac myeloid progenitors, and migrate into the neural folds during early embryogenesis. Before the generation of oligodendrocytes and astrocytes, microglia thus occupy a unique position, constituting the main glial population during early development and participating in a wide array of embryonic and postnatal processes. During this developmental time window, microglia display remarkable features, being highly heterogeneous in time, space, morphology and transcriptional states. Although tremendous progress has been made in our understanding of their ontogeny and roles, there are several limitations for the investigation of specific microglial functions as well as their heterogeneity during development. This review summarizes the current murine tools and models used in the field to study the development of these peculiar cells. In particular, we focus on the methodologies used to label and deplete microglia, monitor their behavior through live-imaging and also discuss the progress currently being made by the community to unravel microglial functions in brain development and disorders.

## Introduction

Microglia, the central nervous system (CNS) resident macrophages remained poorly studied until an exponential growth in interest during the last two decades led to fascinating insights into the origin of microglia, their functions, as well as dysfunctions in pathological conditions (Mosser et al., 2017; Hammond et al., 2018; Hoeffel and Ginhoux, 2018; Li and Barres, 2018; Thion et al., 2018a; Prinz et al., 2019). Contrary to most brain cells, microglia were shown to originate from mesodermal yolk sac (YS) macrophage progenitors that travel to reach the CNS during early embryonic development, around Embryonic day (E)9 in mice and gestational week 4/5 in humans (Monier et al., 2007; Ginhoux et al., 2010; Verney et al., 2010; Menassa et al., 2022). As such, these brain invaders constitute the main glial population before the emergence of other glial cells such as oligodendrocytes and astrocytes. After closure of the blood brain barrier around E14 in mice, microglia are believed to be enclosed in the brain under steady-state conditions. These pioneer microglia thus proliferate, seed the entire parenchyma and progressively mature in symbiosis with the neural tissue microenvironment (Matcovitch-Natan et al., 2016; Thion et al., 2018b; Kracht et al., 2020) before self-renewing throughout life. This situation is different in zebrafish where microglia are fully replaced by another source of microglia by adulthood (Xu et al., 2015; Ferrero et al., 2018). A key aspect of their development is the high heterogeneity in their colonization patterns, morphologies and molecular properties. In particular, microglial colonization of the brain parenchyma is a long-lasting process that spans embryogenesis until the end of the second postnatal week, following a very stereotypical and uneven spatiotemporal pattern (Swinnen et al., 2013; Squarzoni et al., 2014; Menassa et al., 2022). These cells transiently accumulate at specific hotspots such as the cortico-striatal-amygdalar boundary and are excluded from others regions such as the cortical plate. In addition, they display a variety of morphologies (ameboid, poorly ramified, and elongated) associated with different brain localizations. Finally, owing to high throughput approaches, microglia have been shown to exhibit different transcriptomic states, specifically during development, in both mice and humans (Hammond et al., 2019; Li et al., 2019; Sankowski et al., 2019; Kracht et al., 2020). This high developmental heterogeneity contrasts with a relatively uniform distribution in the whole parenchyma alongside homogeneously ramified morphologies and molecular signatures at adult stages. Finally, sex-specific microglial features have been highlighted in postnatal steady-state conditions but also in response to environmental challenges (Schwarz et al., 2012; Lenz et al., 2013; Rebuli et al., 2016; Hanamsagar et al., 2017; Guneykaya et al., 2018; Thion et al., 2018b; Villa et al., 2018; VanRyzin et al., 2019).

Several seminal studies have demonstrated that, beyond their immune functions, microglia also perform a wide array of cellular functions that relate to the normal functioning of the brain and importantly to its development. In particular, they interact with synapses to mediate remodeling, pruning and transmission but have also been involved in synaptogenesis (Andoh and Koyama, 2021). They further participate to neurogenesis and oligodendrogenesis, partly through their regulation of cell death and survival (Sierra et al., 2010; Cunningham et al., 2013; Hagemeyer et al., 2017; Wlodarczyk et al., 2017; Nemes-Baran et al., 2020; Sherafat et al., 2021; Cserep et al., 2022). They also contribute to the refinement of axonal tracts (Pont-Lezica et al., 2014; Squarzoni et al., 2014) and to the development of cortical inhibitory circuits (Squarzoni et al., 2014; Thion et al., 2019; Favuzzi et al., 2021; Yu et al., 2022). Besides, microglia express various pattern recognition, purinergic, chemokine and cytokine receptors, collectively described as the sensome (Hickman et al., 2013), enabling them to detect and integrate environmental changes. Importantly and consistently with their wide array of cellular functions, microglial dysfunction has been associated with the etiology of neurodevelopmental disorders, including autism spectrum disorders and schizophrenia in humans and mouse models (Lukens and Eyo, 2022). Therefore, during this crucial period of development, it is key to better grasp their functions, the regulatory mechanisms underpinning their heterogeneity and how their molecular states may regulate their roles. Furthermore, elucidating how external signals can impact on these fundamental processes is a major challenge. This will be crucial to illuminate specific and diverse microglial contributions to brain wiring as well as shed light on pathological mechanisms of neurodevelopmental disorders.

**FIGURE 1:**
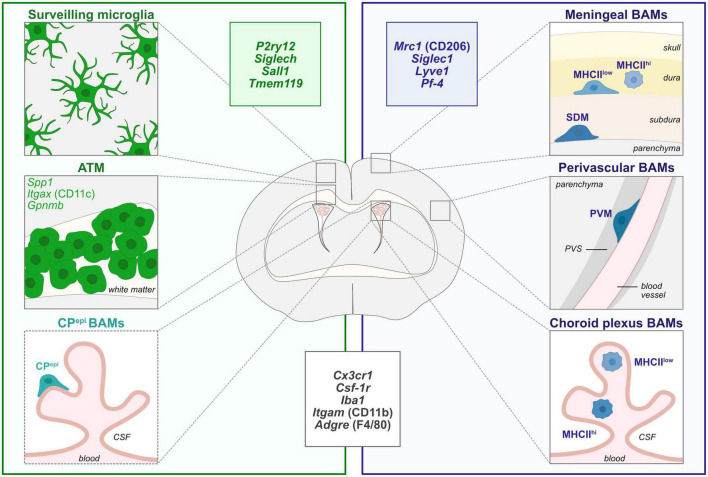
Diversity of CNS macrophages in steady-state conditions. During steady-state, microglia are localized within the brain parenchyma and are highly heterogeneous in morphologies, localization and molecular signatures throughout brain development. In particular, axon tract-associated microglia (ATM)/proliferative-region-associated microglia (PAM)/Cd11c-positive microglia have been described during early postnatal development in the corpus callosum and other white matter regions. Border-associated macrophages (BAMs) reside at distinct interfaces of the CNS such as the meninges, choroid plexus and perivascular space (Goldmann et al., 2016; Mrdjen et al., 2018; Van Hove et al., 2019; Utz et al., 2020; Masuda et al., 2022). Non-parenchymal microglia-like Kolmer’s epiplexus BAMs (CP^epi^) reside in the apical surface of the choroid plexus facing the cerebrospinal fluid (CSF) and share some transcriptional features with microglia and ATM signature (Van Hove et al., 2019). In particular, meningeal MHCII^low^ and MHCII^high^ macrophages are localized in the dura matter while subdural macrophages (SDM) are restrained to the subdural area. Finally, while perivascular macrophages (PVM) are found in the perivascular space, between the vascular basement membrane and the glia limitans of the parenchyma, choroid plexus contains MHCII^low^ and MHCII^high^ BAMs. The insets depict the most commonly used markers for microglia (green), BAMs (blue), and common markers to both populations (black). ATM, axon tract-associated microglia; BAMs, border-associated macrophages; CP^epi^, Kolmer’s epiplexus; CSF, cerebrospinal fluid; MHCII, major histocompatibility complex class II; PVS, perivascular space; PVM, perivascular macrophage; SDM, subdural macrophage.

Beside microglia, other non-parenchymal macrophages called Border-Associated Macrophages (BAMs) are present at the interfaces of the brain: the meninges, choroid plexus and perivascular space (Lee et al., 2021; Figure 1). Although microglia and BAMs originate from yolk-sac derived progenitors and seed the brain during embryogenesis, some of them are further replaced by monocyte-derived cells, arising from hematopoietic stem cells (Goldmann et al., 2016; Mrdjen et al., 2018; Van Hove et al., 2019; Utz et al., 2020; Masuda et al., 2022). Moreover, while generally referred to as BAMs, they display age- and tissue-specific signatures (Kierdorf et al., 2019; Mildenberger et al., 2022). Most of the well-recognized microglial markers, reporter mouse lines and models that currently exist to label and deplete microglia can also target a large part of macrophages such as the BAMs in the CNS but also populations of peripheral macrophages (Green et al., 2020). Consequently, despite intense research efforts, these limitations prevent the identification and characterization of specific microglia functions, especially during development. Herein, we discuss about the current murine tools and models available to label or deplete microglia and subsequently assess their developmental functions in physiological and disease conditions.

## From broad microglial targeting to specific states labeling

### Catch me if you can: Microglia and other brain macrophages

Historically, microglia were mainly identified either through Iba1 immunostaining or using the *Cx3cr1*^*GFP*/+^ (Jung et al., 2000) that labels many macrophages, including microglia and BAMs. Similarly, the well-established *Cx3cr1*^*creERT*2^ mouse lines have been very useful to inactivate genes in these cells (Parkhurst et al., 2013; Yona et al., 2013). Nevertheless, they are not specific to microglia and may trigger microglial reactivity in neonates upon tamoxifen administration (Sahasrabuddhe and Ghosh, 2022). Recent studies, including single cell RNA sequencing analyses, highlighted more specific homeostatic microglia markers including *p2ry12*, *Sall1*, *Tmem119*, *Hexb*, *Siglech* (Gautier et al., 2012; Buttgereit et al., 2016; Satoh et al., 2016; Cserep et al., 2020; Masuda et al., 2020) allowing the use of specific antibodies to label either microglia (P2ry12, Tmem119, and SiglecH) or BAMs (CD206, Lyve1, and Siglec1) (Mrdjen et al., 2018; Figure 1). Fluorescent *In Situ* Hybridization (FISH) has also been used to circumvent the absence of specific antibodies, particularly by taking advantage of the RNAscope technique (Matcovitch-Natan et al., 2016; Hammond et al., 2019). To specifically visualize, manipulate and assess microglia functions, reporter and *creERT2*-expressing lines were generated with minor recombination in BAMs: *Tmem119*^*eGFP*^ and *Tmem119*^*creERT*2^ (Kaiser and Feng, 2019), *Tmem119^tdTomato^* (Ruan et al., 2020), *Hexb^tdTomato^* and *Hexb*^*creERT*2^ (Masuda et al., 2020), *p2ry12*^*creERT*2^ (McKinsey et al., 2020), *Sall1*^*GFP*^, and *Sall1*^*creERT*2^ (Buttgereit et al., 2016), the latter recombining in neurons and other glia (Chappell-Maor et al., 2020; Table 1). Nonetheless, since most of these genes start to be expressed as microglia mature (Bennett et al., 2016), it is important to stress that there are so far no alternatives to the *Cx3cr1*^*GFP*/+^ or *Cx3cr1*^*creERT*2^ lines to efficiently target early embryonic microglia.

**TABLE 1:** Tools to label, target and deplete microglia and BAMs.

Tagging models	Microglia	BAMs	Prenatal and early postnatal	Adulthood	Remarks	References
*Cx3cr1^GFP/+^*	Yes	Yes	GFP expressed by E9.5	>99%	Targets peripheral immune cells and myeloid BM progenitors	Jung et al., 2000
*Cx3cr1^creERT2^* (1,2)	Yes	Yes	40H-Tam. E9.0: 99% efficiency at E10.5 (2)	>99%	Spontaneous recombination reported	Parkhurst et al., 2013 (1); Yona et al., 2013 (2)
*Tmem119^eGFP^*	Yes	Few	GFP expressed by P1	>99%	Transiently labels blood vessels at P1	Kaiser and Feng, 2019
*Tmem119^creERT2^*	Yes	Few	Tam. P2, P3 and P4: 90% efficiency by P14	>99%	Targets few CD45+ cells in blood	
*Tmem119^tdTomat^*	Yes	Few	N.A.	>95%		Ruan et al., 2020
*Hexb^tdTomato^*	Yes	Few	TdTomato expressed by E12.5	>99%	Labels few meningeal and perivascular BAMs	Masuda et al., 2020
*Hexb^creERT2^*	Yes	Few	Tam. P1 and P3: 90% efficiency at P42	^~^80%	Targets peripheral macrophages in kidney	
*P2ry12^creERT2^*	Yes	Few	Tam. E13.5, E15.5 and E17.5: robust efficiency by E18.5 in microglia and subsets of BAMs (40% in choroid plexus, 10% in meninges, few PVM)	90–95%	Targets 20–25% of BAMs in choroid plexus and meninges	McKinsey et al., 2020
*Sall1^GFP^*	Yes	No	GFP expressed in 20% microglia by E12.5, 69% by E14.5, 90% by P2	>95%	Targets <10% other CNS cells	Buttgereit et al., 2016; Utz et al., 2020
*Sall1^creERT2^*	Yes	No	Tam. E14.5 and E16.5: 75% efficiency at E18.5	>95%	Targets <10% other CNS cells	
*Cx3cr1*^ccre^*:Sall1*^ncre^**	Yes	No	N.A.	^~^90%		Kim et al., 2021
*Cx3cr1*^ccre^*:Lyve1*^ncre^**	No	Yes	N.A.	20% of Lyve1+ cells		
*Lyve1^CreERT2^*	No	Yes	40H-Tam. E16.5: 50% efficiency in meningeal and perivascular macrophages at P14	N.A.	Targets lymphatic endothelial cells	Masuda et al., 2022
*Siglec1^cre^*	No	Yes	Efficiently floxes gene in BAMs at E18.5	N.A.	*Siglec1* expressed by 60% BAMs at E14.5, 100% at E18.5	Utz et al., 2020
*Pf4-Cre*	Few	Yes	N.A.	>99%		McKinsey et al., 2020
*Mrc1^CreERT2^*	No	Yes	40H-Tam. E9.0: 10% efficiency in BAMs and 5% in microglia by E18.5	>95%		Masuda et al., 2022
Adenoviruses	Yes	N.A.	N.A.	80%		Lin et al., 2022
Depletion models	Microglia	BAMs	Prenatal and early postnatal	Adulthood	Remarks	References
**Killing by numbers**						
*Cx3cr1*^CreER^*:R26*^iDTR^**	Yes	Yes	N.A.	>99%	Fast repopulation	Parkhurst et al., 2013
*IBA1-tTA::*^DTAtetO/tetO^**	Yes	Yes	Withdrawal of doxycycline from maternal diet from P5: 50% depletion at P8	^~^90% in retina	Fast repopulation	Miyamoto et al., 2016; Takeda et al., 2018
*Siglech^DTR/DTR^*	Yes	No	Injection at E10.5: 80% depletion at E12.5, 60% at E14.5	80–85%	Fast repopulation	Konishi et al., 2017; Li et al., 2021
**CSF-1R inhibitors**						
Anti CSF-1R antibodies	Yes	Yes	Injections at E6 and E7: >98% depletion at E14.5	No effect	Fast repopulation	MacDonald et al., 2010; Squarzoni et al., 2014
Anti CSF-1 antibodies	Yes	No	Injections at E6 and E7: >50% depletion at P0.5	60% in white matter region	Dose-dependent efficiency	Easley-Neal et al., 2019
Anti IL-34 antibodies	Yes	No	Injections at E6 and E7: no effect at P0.5. Injection at P0.5: 30% depletion at P4	50% in grey matter region	Dose-dependent efficiency	
PLX5622	Yes	Yes	Chow PLX from E3.5: 99% depletion at E15.5	>95% within 7 days	Fast repopulation	Huang et al., 2018; Rosin et al., 2018; Marsters et al., 2020
PLX3397	Yes	Yes	Chow PLX from E14 followed by s.c. injections in pups: 90% depletion at P5 in spinal cord	>99% within 7 days	Fast repopulation	Elmore et al., 2014; Li et al., 2020
**Genetic models**						
*Pu.1^–/–^*	Yes	Yes	100%	100%	Homozygotes die shortly after birth	McKercher et al., 1996
*Csf1^op/op^*	Yes	Yes	N.A.	0–30%	Abnormal brain development	Michaelson et al., 1996
*Il34^LacZ/LacZ^* (1,2)	Yes	N.A.	>80% decrease at P2 (1); normal colonization of the brain from E10.5 to newborn (2)	50% (1; 2)		Wang et al., 2012 (1); Greter et al., 2012 (2)
*Csf1r* ^–/–^	Yes	Yes	>99%	100%	Shortened lifespan and abnormal brain development	Erblich et al., 2011
*Sall1*^CreER^*;Csf1r*^fl/fl^**	Yes	No	N.A.	70–90%	Spatial variability in efficiency	Buttgereit et al., 2016
*Hexb*^CreERT2^*;Csf1r*^fl/fl^**	Yes	No	N.A.	60%		Masuda et al., 2020
*Csf1r* ^Δ*FIRE*/Δ*FIRE*^	Yes	Few	Absence of CPepi in the choroid plexus, other BAMs reduced	100%		Rojo et al., 2019; Munro et al., 2020
*Nestin^cre^*;*Csf1^fl/fl^*	Yes	N.A.	60% decrease in E17.5 cerebellum	^~^50% in cerebellum		Kana et al., 2019
*Nestin^cre^*;*Il34^fl/fl^*	Yes	N.A.	N.A.	^~^85% in striatum		Badimon et al., 2020

Table summarizing the main mouse lines and tools available to label, target and deplete microglia and BAMs, indicating their specificity and efficiency in development and adulthood. BAMs, border-associated macrophages; BM, bone marrow; CNS, central nervous system; CPepi, Kolmer’s epiplexus; DTA, diphtheria toxin fragment A; DTR, diphtheria toxin receptor; iDTR, inducible diphtheria toxin receptor; i.p., intraperitoneal; N.A., non-applicable; PVM, perivascular macrophages; s.c., subcutaneous; Tam, tamoxifen; 40H-Tam, 4-hydroxytamoxifen.

Targeting microglia more specifically is crucial, since studies often assign a variety of roles to microglia using depletion models that target both microglia and BAMs. Kim et al. (2021) took advantage of an elegant “split cre” binary genetic construct to generate the *Cx3cr1*^ccre^*:Sall1*^ncre^** and *Cx3cr1*^ccre^*:Lyve1*^ncre^**, to selectively target microglia and BAMs, respectively. Furthermore, several mouse lines including the *Siglec1^cre^* (Utz et al., 2020), *Pf4-Cre* (McKinsey et al., 2020), *Lyve1*^*creERT*2^, and *Mrc1*^*CreERT*2^ lines (Masuda et al., 2022) have been shown to specifically label BAMs during development and thereby enable to follow their trajectory (Table 1). In addition, while no circulating cells are thought to enter in the brain parenchyma during steady-state after BBB closure, monocyte infiltration can occur in disease, aging and injury, and can be monitored using bone marrow chimeras (Mills et al., 2022). These new tools will be important to decipher the relative contributions of microglia, BAMs or infiltrating myeloid cells, but still require thorough characterization with regards to efficiency and rate of spontaneous recombination at different timepoints along development.

Finally, cell-specific viral gene delivery has been extensively used to target different CNS population but robust transduction in microglia remained quite inefficient until recently (Maes et al., 2019). In a ground-breaking study, Lin et al. (2022) successfully targeted 80% of microglia *in vivo* using adeno-associated viruses without inducing microglia reactivity or changes in gene expression, although it remains elusive to what extend BAMs were also affected. Such approach opens new avenues to study microglia but also have tremendous translational potential.

### Looking with new eyes

Along with new markers, mouse lines and viral approaches to target microglia, technical advances in diverse fields shed new light on ways to study microglia, in particular going from fixed immobile imaging in brain slices to dynamic and global approaches. First, tissue clearing methods are constantly improving, with some of them perfectly preserving the signal from reporter lines and antibodies [reviewed in Eme-Scolan and Dando (2020)]. This allows visualization of microglia in whole intact brains. However, while these techniques are becoming well-established and easier to use routinely, the difficulty lies in the analysis of the generated data. Annotated 3D atlases of the developing brain will offer many exciting possibilities toward a more comprehensive study of microglia development.

Another revolution in the field of microglia came with two-photon live-imaging experiments, which revealed never resting microglia with their processes constantly surveilling their environment as well as rapidly reacting in case of injury (Davalos et al., 2005; Nimmerjahn et al., 2005). In combination with other markers, it is thus possible to monitor microglial interaction with blood vessels (Csaszar et al., 2022), radial glia (Rosin et al., 2021), neuronal populations as well as track microglial processes and their specific contact with synapses (Wake et al., 2009) or nodes of Ranvier (Ronzano et al., 2021). While some experiments are performed on brain slices, inducing tissue damage and possibly altering microglial behavior, most studies use cranial windows or skull thinning that allows microglial observation in their homeostatic environment. For pups, adapted approaches are being developed taking advantage of the thinness of the embryonic skull to perform *ex utero* live-imaging of microglia and macrophages in the brain of intact embryos (Hattori et al., 2020, 2022; Munz et al., 2022), that will probably be critical to better characterize key aspects of microglia development such as their entry in the brain parenchyma (Stremmel et al., 2018).

As a self-renewing population arising from a restricted pool of pioneer cells, the questions of microglial expansion during development, their migration and turnover were raised–and still retain some mystery. The “Microfetti” mice developed by Tay et al. (2017) in which microglia express randomly one out of four fluorophores after tamoxifen induction, highlighted clonal expansion of adult microglia in pathology. In addition, Ratz et al. (2022) developed the new TREX technique, which combines single-cell and spatial transcriptomic coupled to early (E9.5) *in vivo* barcoding, in order to analyze the lineage relationships between mature cells and progenitors. Thereby, they highlighted drastic microglial expansion from a limited pool of progenitors. Though not extensively used yet, these tools for clonal analysis and lineage tracing will be valuable in the context of development to better understand microglial expansion, migration and final distribution in the brain.

### Targeting the different flavors of microglia

Historically, microglia were classified as displaying either a neurotoxic pro-inflammatory M1 or neuroprotective anti-inflammatory M2 phenotype but this binary perspective has been largely revisited (Paolicelli et al., 2022). The development of high throughput technologies such as single cell and single nucleus RNA sequencing (sc/snRNA-seq), cytometry by time-of-flight (CyTOF), or multiplex error-robust fluorescent *in situ* hybridization (MERFISH) revealed a richer heterogeneity in microglial profiles. While microglia constitute a relatively homogeneous population in adulthood, they display a striking heterogeneity during prenatal/early postnatal development, aging and neurodegeneration (Deczkowska et al., 2018; Hammond et al., 2019; Li et al., 2019; Masuda et al., 2019; Sankowski et al., 2019; Kracht et al., 2020; Marschallinger et al., 2020; Safaiyan et al., 2021; Stogsdill et al., 2022). Nevertheless, we should not underestimate the experimental bias introduced by cell dissociation and sorting-strategies, sequencing technologies and subsequent analyzes selected to characterize microglial heterogeneity (Marsh et al., 2022; Paolicelli et al., 2022; Sankowski et al., 2022). To avoid extensive confusion in the field, it remains crucial to be cautious about the semantic implication of microglial heterogeneity and the subsequent functional diversity associated with it (Paolicelli et al., 2022).

Specific developmental microglial states are thus starting to be described such as the Axon Tract-associated Microglia (ATM), also known as Proliferative-region-Associated Microglia (PAM), Cd11c-positive microglia, and Youth-Associated Microglia (YAM). They are characterized by the expression of several genes such as *Spp1, Itgax, Gpnmb* and were initially identified in the postnatal white matter as regulators of the development of oligodendrocyte precursors and of subsequent myelinogenesis (Hagemeyer et al., 2017; Wlodarczyk et al., 2017; Hammond et al., 2019; Li et al., 2019; Silvin et al., 2022). Interestingly, there are remarkable similarities in transcriptomic signatures between ATM and Disease Associated Microglia (DAM), initially characterized in mouse models of Alzheimer’s disease (Keren-Shaul et al., 2017; Krasemann et al., 2017), raising the question of their potential similarities, differences–and relationship. Apart from the use of specific antibodies or *in situ* hybridization probes, the field lacks genetic tools to specifically label and target microglia states. So far, only a *CD11c^cre^* line has been used to target ATM (Wlodarczyk et al., 2017), but was unsuccessful in depleting ATM in combination with Diphtheria Toxin strategies. In addition, a novel *Spp1^tdTomato^* mouse line has been generated and used to monitor SPP1 from perivascular macrophages (De Schepper et al., 2022), and could constitute an interesting tool for ATM-lineage studies. Based on their emerging molecular characterization, novel mouse lines or viral approaches will enable specific depletion or inactivation of microglial states to further assess their fates and functions. Moreover, in-depth characterization of transcriptomic, epigenomic, and metabolomic landscapes in microglia would enable to better understand their regulatory mechanisms, the transientness of these states and to which extent they can be induced by the microenvironment at different stages of life. Along these lines, the progress of spatial transcriptomics toward increased structural resolution should lead to a tremendous breakthrough to characterize microglial heterogeneity (Stogsdill et al., 2022), in particular at hotspots of accumulating microglia during development. Altogether, the field is moving onto a specific targeting of developmental microglial states which should shed new light on their regulatory mechanisms, their plasticity and functions in steady-state and disease conditions.

## En route for specific depletion approaches

Microglial functions in brain development, homeostasis and diseases were historically assessed using *in vivo* depletion strategies summarized in Table 1. Though a lot of techniques have been developed, their diversity illustrates the difficulty to obtain a specific, efficient and long-lasting depletion of microglia while limiting its off-target effects.

### Killing by numbers

The first strategies employed were aiming to directly trigger microglial apoptosis by administration of clodronate liposomes, that are specifically phagocytosed by microglia and BAMs and induce cell death upon cytoplasmic release (Green et al., 2020). This approach is efficient in the early post-natal brain and in adults, but the inability of the clodronate liposomes to cross the blood brain barrier requires an intracerebral injection, inducing an injury and possible release of inflammatory cytokines (Han et al., 2019). Another approach uses the diphtheria-toxin (DT) based systems, either with direct expression of the DTA or with DT receptor, the latter needing administration of DT. The first studies were performed in *Cx3cr1*^CreER^*:R26*^iDTR^** and *Iba1-tTA:*^DTAtetO/tetO^** targeting both microglia and BAMs (Parkhurst et al., 2013; Miyamoto et al., 2016; Takeda et al., 2018). On the contrary, the use of the *Siglech^dtr/+^* mice led to specific and transient depletion of embryonic microglia, without affecting BAMs (Li et al., 2021). Thus, the constitutive or inducible DTR/DTA expression, combined with specific microglial lines provides a better temporal control as well as selective effect, though their efficiency remains variable.

### CSF-1R inhibitors

The CSF-1 receptor (CSF-1R) is expressed by microglia, macrophages and their progenitors and its signaling is essential for their survival and proliferation. It thus became a preferential target in the quest for microglial depletion methods. Injections of an anti-CSF-1R antibody performed at E6.5 and E7.5 lead to a drastic depletion of myeloid progenitors, macrophages and microglia, while repopulation spans the first postnatal week (Squarzoni et al., 2014; Hoeffel and Ginhoux, 2015; Thion et al., 2019). On the other hand, embryonic targeting of CSF-1, one of the two CSF-1R ligands, through anti-CSF-1 antibody, leads to a drastic depletion of forebrain microglia (Easley-Neal et al., 2019), with possible off-target effects of the circulating antibodies. Pharmacological inhibitors of the CSF-1R injected or delivered non-invasively *via* food pellets have been broadly developed to deplete adult microglia, such as PLX3397 and PLX5622, the latter having a higher specificity and improved brain penetrance (Elmore et al., 2014; Green et al., 2020) while it efficiently depletes embryonic microglia, enabling a temporal control of the depletion during gestation (Rosin et al., 2018; Marsters et al., 2020). Nevertheless, as this treatment can affect lactation by depleting maternal macrophages essential for mammary gland development (O’Brien et al., 2012), it can impact pup survival following birth. For early postnatal depletion, direct subcutaneous injections of PLX3397 or PLX5622 during the first post-natal week allows for an efficient depletion of microglia (Li et al., 2020; Favuzzi et al., 2021; Gesuita et al., 2022). Although beyond the scope of this review, CSF-1R inhibitors are also used in other species including humans, bringing novel therapeutic approaches [reviewed in Han et al. (2022)]. Overall, targeting the CSF-1R pathway has proven to be efficient and convenient, but also affects peripheral macrophages and BAMs, preventing the identification of specific microglial functions.

### Genetic models

Different genetic models, including constitutive knock-outs of fundamental transcription or survival factors, like *Pu.1^–/–^* (McKercher et al., 1996) and *Csf1r*^–/–^ (Erblich et al., 2011), fail to develop microglia as well as most macrophages (Green et al., 2020). This results in many off-target effects and early death in *Pu.1^–/–^* and *Csf1r*^–/–^. On the other hand, the *Csf1r^fl/fl^* allows for a more specific targeting of microglia, thanks to the microglia specific mouse lines now available and their possible temporal induction. This strategy has been used with *Sall1*^CreER^*;Csf1r*^fl/fl^** (Buttgereit et al., 2016) and *Hexb*^*CreERT*2^;*Csf1r*^*fl/fl*^ mice (Masuda et al., 2020), with a respective efficiency varying across brain regions from 70% to 90% and of 60%.

An additional important model is the *Csf1r*^Δ*FIRE*/Δ*FIRE*^ mice, in which a *Csf1r* enhancer is deleted (Rojo et al., 2019; Munro et al., 2020). This leads to the absence of microglia from the brain parenchyma, and of resident macrophages of the skin, kidney, heart and peritoneum; while other macrophages and monocytes are unaffected. Importantly, these mice are healthy and fertile in a mixed B6CBAF1/C57BL/6J background, and do not display strong developmental defects described in the *Csf1r*^–/–^. So far, they were used to investigate the role of resident microglia in Alzheimer disease mice (Kiani Shabestari et al., 2022) and they will provide a more specific and long-lasting model to explore microglial functions.

Finally, full inactivation of CSF-1R ligands, *Csf-1* (Chang et al., 1994; Kondo and Duncan, 2009) or *Il34* (Greter et al., 2012; Wang et al., 2012), expressed by the neural tissue, results in time- and region-dependent partial depletion of microglia albeit there are still some controversies amongst studies and major impact on peripheral macrophages (Green et al., 2020; Table 1). Nevertheless, under the pan neuronal driver *Nestin^cre^* that restricts depletion to the nervous system, thus mainly affecting microglia, the *Nestin^cre^*;*Csf1^fl/fl^* and *Nestin^cre^*;*Il34^fl/fl^* are respectively deprived of microglia in the white matter and cerebellum or in the gray matter highlighting region-specific dependency (Kana et al., 2019; Badimon et al., 2020). On the other hand, macrophages are unaffected in the yolk sac, fetal liver and fetal limbs. Last, because the CSF-1R ligands are differentially expressed and required from microglia through development, these lines provide promising models to investigate local microglial functions.

## Concluding remarks

Microglia now appear as key actors of brain development, interacting with most brain cells and regulating several crucial developmental processes. Nevertheless, these exciting advances were done in models affecting both microglia and BAMs. Thus, dissecting their respective contributions thanks to new specific tools will be key to the field. In addition, further understanding microglial diversity during development and the functions played by specific states should bring new light on their importance in neurodevelopmental pathologies and open new avenues for therapeutic intervention.

## Author contributions

CB and MST wrote the review. Both authors contributed to the article and approved the submitted version.
